# S-Amlodipine: An Isomer with Difference—Time to Shift from Racemic Amlodipine

**DOI:** 10.1155/2018/8681792

**Published:** 2018-05-20

**Authors:** Jamshed Dalal, J. C. Mohan, S. S. Iyengar, Jagdish Hiremath, Immaneni Sathyamurthy, Sandeep Bansal, Dhiman Kahali, Arup Dasbiswas

**Affiliations:** ^1^Centre for Cardiac Sciences, Kokilaben Dhirubhai Ambani Hospital, Mumbai, India; ^2^Fortis Hospital, Shalimar Bagh, New Delhi, India; ^3^Manipal Hospital, Bangalore, India; ^4^Ruby Hall Clinic, Pune, India; ^5^Apollo Hospitals, Chennai, India; ^6^Department of Cardiology, VMMC & Safdarjung Hospital, New Delhi, India; ^7^B M Birla Heart Research Centre, Kolkata, India; ^8^NRS Medical College and Hospital, Kolkata, India

## Abstract

Calcium channel blockers are among the first-line drugs for treatment of hypertension (HTN). S-amlodipine (S-AM), an S-enantiomer of amlodipine, is available in India and in other countries like China, Korea, Russia, Ukraine, and Nepal. Being clinically researched for nearly two decades, we performed in-depth review of S-AM. This review discusses clinical evidence from total 42 studies (26 randomized controlled trials, 14 observational studies, and 2 meta-analyses) corroborating over 7400 patients treated with S-AM. Efficacy and safety of S-AM in HTN in comparison to racemic amlodipine, used as monotherapy and in combination with other antihypertensives, efficacy in angina, and pleiotropic benefits with S-AM, are discussed in this review.

## 1. Introduction

Management of hypertension (HTN) involves different therapeutic approaches. Among the medications for treating HTN, calcium channel blockers (CCBs) are one of the first-line agents as recommended by recent Joint National Committee 8 (JNC-8) guidelines [1]. Besides efficacy, occurrence of adverse effects (AEs) plays an important role in maintaining adherence with medications [2]. Occurrence of peripheral edema is the major reason for poor adherence with amlodipine. The Anglo-Scandinavian Cardiac Outcomes Trial-Blood Pressure Lowering Arm (ASCOT-BPLA) [3] reported peripheral edema in 23% patients receiving amlodipine. This suggests that nearly 1 out of 4 patients treated with amlodipine may develop peripheral edema. Conventionally used amlodipine is a mix of S- and R-enantiomers. Development of separate enantiomers improves pharmacokinetics (PK) and avoids undesirable AEs [4]. From R- and S-isomers of amlodipine, S-enantiomer has nearly 1000 times greater affinity for the receptor site. Further, S-amlodipine (S-AM) has less variable PK, lower intrasubject variation, and longer half-life [5]. S-AM is equally efficacious at half-dose with better tolerability and lesser incidence of peripheral edema than racemic amlodipine (Amlo) [6].

S-AM is marketed world-wide. The central drugs standards control organization (CDSCO), India, approved S-AM on 16 August 2002 for its use in HTN [7]. Globally, S-AM has been approved and is being used in countries like China [8], Korea [9], Ukraine [10], Philippines [11], and Nepal [12]. Besides these, S-AM is marketed in nearly 47 countries [13]. Since S-AM approval in China (1999) [14] and in India (2002) [7], it has been studied extensively. As being researched for nearly two decades, we performed an in-depth review of clinical evidence of S-amlodipine and provided key summary with identification of areas for further research.

## 2. Search Methodology and Literature Details

We performed search using terms “S-Amlodipine” or “levamlodipine” across electronic databases like PUBMED, Google Scholar, and clinical trials registry, http://www.clinicaltrials.gov. Additionally, a general search at Google search engine was performed. Clinical studies including randomized trials and observational and postmarketing studies before June 2017 were included in the review. Journals articles available only as print copies were also included in the review. For non-English literature articles, information available from the abstracts was captured.

After an extensive search, we included total 42 studies. In these, 26 were RCTs (20 monotherapy and 6 combination studies), 14 were observational studies (13 monotherapy studies and 1 combination study) and two were meta-analyses. From these, a maximum number of studies (*n* = 18) were from China followed by 11 from India, 6 from Korea, 3 each from Russia and Ukraine, and one from Sri Lanka. Combined from all the studies, over 7400 patients had received S-AM either alone or in combination with other antihypertensives. In these studies, racemic amlodipine was the major comparator in 26 studies and in two meta-analyses as well. As monotherapy and/or combination therapy, other comparator molecules from 10 studies were lercanidipine (Lercan), nifedipine sustained release (Nifed-SR), cilnidipine (CLD), ramipril (Rami), enalapril (Enala), losartan (Los), telmisartan (Telmi), and indapamide. In five observational studies, there was no comparator to S-AM. In two combination studies, the combination treatment was compared to S-AM monotherapy.

## 3. S-Amlodipine in Hypertension

For its use in HTN, S-AM has been evaluated in various RCTs (total 22) and observational studies (total 9) either as monotherapy (total 25) or in combination (total 6 RCTs only). Two meta-analyses were performed in 2010 and 2015 with 15 and 8 studies of S-AM (levamlodipine), respectively. Major findings from the RCTs, observational studies, and meta-analyses are summarized in Tables 1, 2, and 3, respectively. Most of these studies were comparing S-AM (2.5 to 5 mg) to racemic amlodipine (5 to 10 mg) and found near equal antihypertensive efficacy with lower incidence of side effects. Two RCTs especially evaluated ankle (peripheral) edema with S-AM in comparison to Amlo and reported significantly lower incidence of edema with better tolerability of S-AM [15, 16]. Besides racemic amlodipine, S-AM was compared to lercanidipine [17, 18] and ramipril [19] in three trials. S-AM had nearly similar efficacy and tolerability to lercanidipine. However, its efficacy and safety were better than that of ramipril (Table 1). A study from Chen et al. [20] needs a special mention as they compared higher-dose (5 mg) to the lower-dose (2.5 mg) of S-AM (Table 1). After 8-week treatment, 24-hour ambulatory systolic BP (SBP) reduction was significantly greater in 5 mg group than in 2.5 mg of S-AM (between group difference: 2.1 mmHg, *p* = 0.02). However, 24-hour diastolic BP (DBP) reduction was similar (between group difference: 0.9 mmHg, *p* = 0.17). Interestingly, the incidence of overall AEs was similar (20.0% versus 17.0%, resp., *p* = 0.05) in both groups and proportion of individual AEs was nearly equal in both doses. This perpetuates that S-AM can be safely used of high-dose of 5 mg per day with incremental efficacy.

All nine observational studies were monotherapy trials (Table 2). In these, racemic amlodipine was comparator in four studies, lercanidipine in one, and cilnidipine in one trial. Four studies were single arm trials with no comparator. Four studies without any comparator, the safety and efficacy of S-amlodipine (SESA) studies, were the postmarketing trials that reported significant BP reduction with significantly less or no occurrence of pedal edema in Indian hypertensive patients (Table 2) [21–24]. Occurrence of edema with S-AM in comparison to Amlo and cilnidipine was evaluated in another observational study from India. Incidence of peripheral edema with S-AM and cilnidipine was significantly lower than racemic amlodipine in males (6.7% and 0.0% versus 36.7%, resp.) and in females (10.0% and 3.3% versus 43.3%, resp.) (*p* < 0.001 for both drug comparisons in either gender) (Table 2) [25].

S-AM was also assessed in combination with other antihypertensives like atenolol [26, 27] and telmisartan [28, 29] and in patients receiving both angiotensin converting enzyme inhibitor or angiotensin receptor blocker (ACEI/ARB) and beta blocker (BB) [16]. In studies of combination with atenolol and ACEI/ARB + BB, S-AM had similar antihypertensive efficacy compared to racemic amlodipine (Table 1). However, in two separate studies, combination of S-AM and telmisartan (40–80 mg) was associated with greater BP reduction compared to monotherapy of telmisartan 80 mg or S-AM 2.5 mg (Table 1). Tolerability of telmisartan-based combinations was reported to be similar or better than the comparative monotherapy treatments (Table 1).

In a meta-analysis (Table 3) of 15 trials, Liu et al. [30] reported similar effect of S-AM (2.5 mg) on BP compared to racemic amlodipine (5 mg). From three high-quality RCTs included in the meta-analysis, weighted mean difference (WMD) of SBP and DBP was −2.84 (95% confidence interval (CI), −6.42 to 0.74) and −1.71 (95% CI, −3.48 to 0.06), respectively, after 4-week treatment (one RCT) whereas it was −1.13 (95% CI, −5.29 to 3.03) and −1.34 (95% CI, −2.67 to –0.01), respectively, after 8-week treatment (two RCTs). Further, S-Amlo was associated with significantly less edema than racemic amlodipine (risk difference, –0.02; 95% CI, −0.03 to 0.00). Another meta-analysis performed recently by Zhao and Chen [31] involving 1456 patients from eight studies reported that levamlodipine (S-AM) was efficacious (odds ratio (OR) 2.19, 95% CI 1.61–2.97; *p* < 0.01) and safer (OR 0.51, 95% CI 0.34–0.77; *p* < 0.01) than racemic amlodipine. Thus, available evidence from RCTs, observational studies, and meta-analyses finds equivalent BP lowering efficacy of S-AM against racemic amlodipine with better tolerability. Incidence of pedal edema is found to be significantly lesser with S-AM than racemic amlodipine.

## 4. S-Amlodipine in Angina

The antianginal effects of racemic amlodipine are known. Systemic vasodilation with reduction afterload reducing cardiac workload and dilatation of coronary vasculature and reduction in cardiac oxygen consumption underlie the relief in anginal cases. Being an isomer of amlodipine, S-AM has also shown efficacy in angina. In SESA-Angina study (2005) [32] conducted in India, patients of ischemic heart disease (IHD) with history of angina and positive stress test (*n* = 25) were included. No other concomitant treatments were allowed during the treatment period of 8 weeks. S-AM (2.5–5 mg/d) treatment was associated with significant reduction in average number angina attacks in every 15 days (*p* < 0.0001) and significant improvement in anginal symptoms (94.1%). After treatment, there was significant increase in exercise capacity (*p* < 0.0001) and nonsignificant increase in time required for 1.5 mm ST-segment depression (*p* = 0.1764) and maximum workload achieved (*p* = 0.1170). No AEs were reported in any patient. This emphasizes efficacy and safety of S-AM in management of angina.

## 5. S-Amlodipine and Pleiotropic Benefits

### 5.1. Effect on Arterial Stiffness and Endothelial Function

Efficacy of S-AM for change in arterial stiffness and endothelial function was assessed in four RCTs [33–36] and in one observational study [37]. In a 12-week randomized study, Liangjin et al. (2013) [33] compared levamlodipine (S-AM, 2.5–5 mg, *n* = 40) to nifedipine sustained release (Nifed-SR, 10 mg, *n* = 40) for its effect on BP variety ratio (BPVR) and CIMT. Compared to baseline, systolic and diastolic BPVR was significantly better with S-AM than Nifed-SR at 12 weeks. CIMT was reduced significantly with S-AM (*p* < 0.05) but not with Nifed-SR (Table 4). There was significant correlation of BPVR with CIMT in S-AM group. Changes in lipid parameters and C-reactive protein were nonsignificant in both groups.

One RCT [34] reported significant improvements in flow mediated dilatation [FMD] after 6-week treatment with S-AM and racemic amlodipine. Continued treatment for 12 weeks was found to lower serum cholesterol equally in both groups. Guo et al. [35] reported significant improvements in the central BP components, brachial-ankle pulse-wave velocity (PWV), ambulatory arterial stiffness index (AASI), and the variability of ambulatory BP in both S-AM and racemic amlodipine treatment. However, both treatments were not associated with significant changes in CIMT. Thus, the benefits with S-AM on vascular function are similar to those exerted by racemic amlodipine. In another 6-week, randomized, crossover trial, Si et al. [36] reported that FMD%, nitric oxide (NO) and endothelial nitric oxide synthase (eNOS) levels were significantly improved in both groups with no between treatment differences. Increase in NO levels in cultured human umbilical vein endothelial cells was significant with both treatments but more marked in Amlo. Authors concluded that, with S-AM, probably antihypertensive effect is the cause of improved vascular function and S-AM may exert its protective effect on endothelial function by unknown mechanism. However, a 6-month study which assessed effects of S-AM (5–10 mg/d) and enalapril (10–20 mg/d) combination compared to enalapril alone on endothelial dysfunction in patients with chronic pulmonary heart disease (CPHD) and HTN (*n* = 65) observed that S-AM added to enalapril is associated with further improvements in endothelial function than enalapril alone in HTN. This was probably because of more pronounced reduction in endothelin-1 (ET-1) level after treatment with two drugs (3.86 ± 0.24 to 1.95 ± 0.19 pg/mL, *p* < 0.05) compared to enalapril alone (3.32 ± 0.27 to 1.83 ± 0.21 pg/mL, *p* < 0.05). Therefore, though there remains uncertainty about possible mechanisms, S-AM may exert some protection effect on endothelium by improving eNOS levels and reducing ET-1 levels [37].

### 5.2. Effect on Structure and Function of Left Ventricle and Brachial Artery

Iskenderov and Saushkina (2013) [38] assessed S-AM (*n* = 61) and Amlo (*n* = 66) in stages 1-2 HTN patients using left ventricular (LV) and brachial artery structural and functional parameters. After 24-week treatment, S-AM was associated with comparable BP reduction to Amlo, but the mean dose was significantly lower (7.5 ± 0.8 versus 11.6 ± 1.4 mg/day; *p* < 0.01). Significant improvement in LV structure and function and brachial artery function were reported. Reductions in atherogenic lipoproteins and total cholesterol were also significant with S-AM.

### 5.3. Efficacy in Renal Transplant Patients

Tang et al. (2003) [39] observed that, in kidney transplant patients with HTN (*n* = 20), S-AM (2.5 to 5 mg) treatment for 2 months was associated with significant reduction in SBP (*p* < 0.01), DBP (*p* < 0.01), and blood nitrogen (*p* < 0.05) with no increase of serum creatinine (*p* > 0.05). Normalization of BP was reported in 85% of patients.

### 5.4. Efficacy in Insulin Resistance

In a randomized, double-blind, prospective cohort study in type 2 diabetes (T2D) patients, Xiao et al. [40] compared effects of S-AM (2.5–5 mg/d, *n* = 112) and losartan (50–100 mg/d, *n* = 115) after treatment for 36 months (156 weeks). They had followed patients at first, second, and third year of the study. Difference in the reduction in SBP and DBP at the end of 12 months was statistically significant between two groups. However, there were no significant differences between the groups when assessed at the end of 24 or 36 months. Change in fasting insulin levels (mIU/L) and insulin sensitivity index (ISI) was significant with both S-AM and losartan by the end of 3 years (*p* < 0.05). This establishes equivalent efficacy of S-AM to an ARB, losartan in improvement of insulin sensitivity in patients with HTN and impaired fasting glucose.

### 5.5. Effect on Platelet Aggregation

In patients of HTN and T2D, Li et al. (2013) [41] studied effect of levamlodipine on platelet aggregation and expression of matrix metalloproteinase (MMP) 9 and MMP 2. In 32 patients treated, platelet aggregation maximal assessed by coagulation instrument TYXN-91A reduced significantly (*p* < 0.05) from 47.77 ± 11.92 (pretreatment) to 40.78 ± 13.97 (posttreatment). Platelet inhibition rate was 13.50 ± 25.23%. There was no effect on levels of MMP 9 and MMP 2. This study highlights that S-AM has potential to prevent platelet aggregation in high-risk patients like HTN with T2D.

## 6. S-Amlodipine and Pedal Edema

CCBs are associated with a considerable risk of peripheral oedema that may reduce patient compliance or necessitate switching to a different drug. It has been now well-established that S-AM is associated with lower incidence of pedal edema and improved compliance to therapy as evident from studies discussed above. Of note is a recent RCT from Galappatthy et al. (2016) [16] where the incidence of leg edema was the primary outcome assessed. Patients uncontrolled with BB and ACEI/ARB (*n* = 172) were randomized to S-AM 2.5–5 mg (*n* = 86) and racemic amlodipine 5–10 mg (*n* = 86). With S-AM, absolute risk reduction of new edema was 15.1%, relative risk reduction was 32.47%, and number needed to treat was seven (NNT = 7). In SESA trial, edema was resolved in 98.72% patients after switching from racemate amlodipine to S-AM [21]. In SESA-II study done in 2230 patients with HTN, incidence of pedal edema was reported in 41.90% patients who were taking racemic amlodipine before switching over to S-AM [22]. When patients were switched over to S-AM, resolution of pedal edema was noted in 93.07%. Overall incidence of pedal edema was 1.92% with S-AM and the relative risk reduction of pedal edema after S-AM switch was 95.4%. Thus, the evidence convincingly suggests minimal incidence of edema with S-AM compared to racemate amlodipine. The confirmatory evidence is observed in a meta-analysis of 15 RCT of S-AM where Liu et al. [30] reported that S-AM (*n* = 907) was associated with significantly less edema than racemic amlodipine (*n* = 897) (risk difference [RD], −0.02; 95% CI, −0.03 to 0.00; test for overall effect: *Z* = 2.20; *p* = 0.03).

Higher incidence of pedal edema is likely to result in higher degree of discomfort. Therefore, use of chirally pure S-AM would be advantageous due to lower incidence of edema which could result in improved adherence to therapy and hence optimum BP control. Amlodipine causes mainly precapillary vasodilatation without proportional increase of postcapillary blood flow, which leads to peripheral edema. Although R-amlodipine does not have calcium channel blocking properties, it reduces activity of postural vasopressor reflex, which increases the pressure in capillary vessels that activates egress of fluid into surrounding tissues. Studies have shown that nitric oxide (NO) released by the inducible nitric oxide synthase is responsible for development of edema. R (+) amlodipine is involved in local NO formation through the kinin pathway and this may lead to loss of the precapillary reflex vasoconstriction and development of edema when racemate mixture is used. S-AM at any concentration was not found to release NO and does not affect postural vasopressor reflex [42].

## 7. S-Amlodipine and Cost-Effectiveness

From China, Hu et al. (2014) [43] conducted a retrospective cost-effectiveness analysis from two multicentre RCTs of S-AM (2.5 mg/d, *n* = 110) and Amlo (5 mg/d, *n* = 104). With 4–8 weeks of treatment, efficacy rate of both drugs was similar (84.91% and 77.45%, resp.). Cost figures observed for 1 mmHg reduction with S-AM and Amlo were 8.1 Yuan (~1.2 $) and 10 Yuan (~1.5 $) for SBP and 16.9 Yuan (~2.5 $) and 21.7 Yuan (~3.2 $) for DBP, respectively. Reported AEs were 4.6% and 10.3% in two groups, respectively. Thus, study suggests S-Amlo is more cost-effective than racemic amlodipine.

## 8. Summary

Compared to racemic amlodipine, S-AM had equivalent antihypertensive efficacy* at half-dose*. Evidence suggests efficacy of S-AM in 24-hour ambulatory BP reduction, including day-time and nigh-time BP reduction. It was also found to be effective in nocturnal HTN showing its effectiveness in nondippers. Meta-analyses showed equivalent efficacy of S-AM compared to racemic amlodipine with similar or lower rates of AEs. Significantly lower incidence of peripheral edema suggests a better tolerability of S-AM and absolute risk reduction of 15.1% in peripheral edema is seen. Otherwise, overall incidence of AEs was nearly similar with two treatments. Compared to cilnidipine, incidence of edema was found to be nearly similar with S-AM, whereas it was significantly lesser in both drugs when compared to racemic amlodipine. Higher-dose S-AM (5 mg) was more effective and equally safe as that of lower-dose (2.5 mg). In* combination* with telmisartan, atenolol, and enalapril, S-AM showed greater antihypertensive effect with better safety and tolerability. Besides HTN, S-AM was found effective and safe in angina. It lowers numbers of attacks and improves symptoms. S-AM had shown BP lowering efficacy in renal transplant cases with no significant adverse effect on functional renal parameters.

Besides being potent antihypertensive, S-AM showed various pleiotropic benefits. These include improvement in endothelial function, slowing of CIMT progression or reversal of increased CIMT, improvement in arterial stiffness, regression of LVH and improvement in LV diastolic function, improvement in lipid profile, improvement in insulin sensitivity, and reduction in platelet aggregation.

Analysis from China identified S-AM as the cost-effective therapy with economic savings compared to racemic amlodipine.

## 9. Limitations

Although we did extensive search of literature, there is likely chance of missing on non-English literature not covered under the databases searched. Most of the non-English articles were available as abstracts only.

## 10. Conclusion

An equivalent antihypertensive efficacy to racemic amlodipine with lesser or negligible peripheral edema proves S-amlodipine as a cost-effective treatment option in HTN. It is effective, safe, and well-tolerated in combination with other antihypertensives as well. Besides HTN, its efficacy in angina makes it suitable agent in patient with both comorbidities. Pleiotropic benefits like improvement in endothelial function and insulin sensitivity show its promise in patients with comorbidities like diabetes. Given its positive effects on BP, endothelial function, platelet aggregation, insulin sensitivity, and atherogenic lipids, S-AM is likely to lower the adverse cardiovascular outcomes. The evidence from this review clearly suggests that S-amlodipine may be considered as one of the first-choice antihypertensive in patients with HTN including those with heightened cardiovascular risk. Future research should focus on cardiovascular outcomes with S-AM in patients with HTN and other comorbidities.

## Figures and Tables

**Table 1:** Randomized controlled trials of S-amlodipine in hypertension.

Author (year)	Country	S-AM (mg)	Comparator (mg)	*n*	Duration (weeks)	Antihypertensive efficacy	AEs
*Monotherapy Studies (n = 16)*							

Liu et al. (2001) [44]	China	2.5	Amlo (5)	30/30	4	Equivalent	NA

Fang (2002) [45]	China	2.5	Amlo (5)	140/140	40	Equivalent	NR

Cheng et al. (2002) [46]	China	2.5–5	Amlo (5–10)	60/60	5	Equivalent	No difference, milder with S-AM

Hiremath and Dighe (2002) [47]	India	2.5	Amlo (5)	25/25	6	Mean change of SBP/DBP (S-AM Vs Amlo) Standing: −24.21/−13.08 vs −21.6/−13.44 Supine: −27.13/−14.17 vs −22.04/−13.68 Sitting: −26.86/−14.17 vs −23.06/−14.28	None

Kerkar (2003) [48]	India	2.5	Amlo (5)	25/25	6	Mean change of SBP/DBP (S-AM Vs Amlo) Standing: −22.6/−12.72 vs −21.96/−13.24 Supine: −22.84/−13.18 vs −22.32/−13.4 Sitting: −20.16/−13.76 vs −22.24/−15.0	None

Pathak et al. (2004) [49]	India	2.5	Amlo (5)	97/91	6	Mean change of SBP/DBP (S-AM Vs Amlo) Standing: −19.22/−13.63 vs −19.14/−12.76 Supine: −19.69/−13.95 vs −19.24/−13.33 Sitting: −19.87/−14.31 vs −19.24/−13.05	None

Zhang (2006) [50]	China	2.5–5	Amlo (5–10)	36/36	8	S-AM: 165.30/98.22 to 132.70/81.87 Amlo: 164.30/99.30 to 134.10/85.61	Number: 1 vs 6

Bae et al. (2008) [51]	Korea	2.5	Amlo (5)	58/60	8	SBP: −24.27 ± 11.55 vs −25.24 ± 12.47 DBP: −14.73 ± 8.9 vs −14.56 ± 9.28 S-AM non-inferior to Amlo	No significant differences

Zhu et al. (2008) [52]	China	2.5–5	Amlo (5–10)	44	8	Mean change in SBP: 156.26 to 131.50 vs 158.23 to 131.74 DBP: 98.48 to 83.28 vs 99.18 to 83.19	None

Youn et al. (2010) [17]	Korea	2.5	Lercani	32/29	8	Mean change sSBP: −20.5 ± 13.6 vs −19.93 ± 14.5 sDBP: −14.03 ± 8.07 vs −12.93 ± 8.68	None

Kim et al. (2011) [19]^#^	Korea	2.5	Rami (2.5–5)	68/70	8	Mean change SBP: −18.1 ± 7.91 vs −14.3 ± 11.96 (*p* = 0.047) DBP: −12.7 ± 7.02 vs −9.6 ± 7.38 (*p* = 0.023) BP normalization rate: 81.3% vs 61.4% (*p* = 0.017)	5.8% vs 14.2% (*p* = 0.012)

Shengye (2012) [53]	China	2.5–5	Amlo (5–10)	90/90	8	Equivalent	Milder with S-AM

Oh et al. (2012) [15]	Korea	2.5–5	Amlo (5–10)	17/17^*∗*^	12	Mean change sSBP: −21.82 ± 8.76 vs −26.82 ± 11.89 (*p* = 0.172) sDBP: −14.71 ± 6.94 vs −10.88 ± 5.81 (*p* = 0.091)	Significant improvement in ankle edema with S-AM (AFV difference: −70.26 mL, *p* = 0.028)

Zhao (2013) [54]	China	NA	Nifed-SR	61/61	NA	Significant reduction in BP in both groups Overall response rate: 91.8% vs 80.33% (*p* < 0.05)	Lower with S-AM: 6.56% vs 18.03% (*p* < 0.05)

Parvathi et al. (2014) [55]	India	2.5	Amlo (5)	54/54	12	SBP change: −32.4 vs −26.9 DBP change: −13.4 vs −12.0	Edema significantly lower with S-AM: mean change AC: 0.26 vs 0.02 (*p* < 0.009)

Chen et al. (2017) [20]	China	2.5	S-AM (5)	263/260	8	SBP: 6.0 vs 8.1 (*p* = 0.02) DBP: 3.8 vs 4.7 (*p* = 0.17) Target BP achievement: SBP: 81.8% vs 90.8% DBP: 84.0% vs 94.2% SBP&DBP: 75.7% vs 87.3% (*p* ≤ 0.003)	17.0% vs 20.0 (*p* = 0.05)

*Combination Studies (n = 6)*							

Rajanandh et al. (2013) [26] [+Atenolol 50 mg]	India	2.5	Amlo (5)	32/32	24	Mean change SBP: 40 vs 40 DBP: 28 vs 34 Non-significant difference	No difference: 21.9% vs 31.3%

Maksimova et al. (2013) [27] [+Atenolol]	Russia	2.5	Amlo (5)	Total: 31		LSM reduction SBP: −15.9 vs −12.7 DBP: −7.3 vs −5.3 HR: −3 vs −4	Number: 8 vs 16

Hu and Xiao (2013) [56] [S-AM + Irbesartan vs Indapamide + Irbesartan]	China	NA	Indapamide	83	12–24	At 12 and 24 weeks Lower 24-h DBPV, day-time SBPV with S-AM than Indapamide At 24 weeks Significantly lower morning SBP, 24-h DBPV and SBPV	NA

Ihm et al. (2016) [28] [Telmisartan + S-AM FDC (CKD-828): 2.5/40 and 2.5/80]	Korea	2.5	S-AM 2.5	63/63/61	8	Mean NP change in groups: 2.5/40 & 5/40 vs S-AM 2.5 SBP: −12.89, −13.79 vs −4.55 DBP: −9.67, −10.72 vs −4.93 Differences in mean change: SBP: −4.34 (*p* = 0.0028) and −5.61 (*p* < 0.0001) DBP: −4.73 (*p* = 0.0002) and −5.79 (*p* < 0.0001) Achieving BP <140 or <90 mmHg: 60.32% (*p* = 0.0004), 60.66% (*p* = 0.0003) vs 28.33%	9.52% (*p* = 0.0086), 14.29% (*p* = 0.0632) vs 27.87%

Park et al. (2016) [29] [Telmisartan + S-AM FDC: 2.5/40 and 2.5/80 mg]	Korea	2.5 & 5	T (80)	61/60/62	8	2.5/40 & 5/40 Vs T80 SBP: −10.56, −12.32 vs −2.44 DBP: −8.12, −9.58 vs −1.76 (*p* < 0.0001 for both) Achieving target BP <140 or <90: 35.59%, 40.68% vs 11.86%	No differences: 18.6%, 20.0% vs 22.6%

Galappatthy et al. (2016) [16] [ACEI/ARB + BB]	Sri Lanka	2.5–5	Amlo (5–10)	76/70	16	Responders Rate: Similar- 98.57% vs 98.68%	New pitting edema: 31.40 vs 46.51% (*p* = 0.0301); ARR: 15.1% Increase in pitting edema score (*p* = 0.038) & patient rated edema score (*p* = 0.036) with Amlo

AC: ankle circumference, ACEI/ARB: angiotensin converting enzyme inhibitor/angiotensin receptor blocker, AEs: adverse effects, AFV: ankle-foot volume, Amlo: racemic amlodipine, ARR: absolute risk reduction, AT: atenolol, BB: beta blocker, BP: blood pressure, DBP: diastolic BP, DBPV: DBP variability, FDC: fixed-dose combination, HR: heart rate, LSM: least square mean, Nifed-SR: nifedipine sustained release, NA: not available, NR: not reported, RRR: relative risk reduction, RR: risk reduction, S-AM: S-amlodipine, SBP: systolic BP, SBPV: SBP variability, and T: telmisartan; ^*∗*^only females; ^#^chlorthalidone 12.5 mg was added to treatments if BP remained uncontrolled with study medications.

**Table 2:** Observational studies of S-amlodipine in hypertension.

Author (year)	Country	S-AM (mg)	Comparator (mg)	*n*	Duration (weeks)	Antihypertensive efficacy	AEs
SESA (2003) [21]	India	2.5–5	-	1859 2.5: 1514 5: 345	4	2.5 mg: 161/100 to 129/84 5 mg: 179/107 to 137/86 *p* < 0.0001 for both	Rate: 1.61% Edema: 0.75%

SESA-II (2005) [22]	India	2.5–5	-	2230	4	SBP: −26.65 DBP: −13.30 HR: −3.51	Reduction in pedal edema: 93% cases, RRR: 95.4%

SESA-IV (2007) [23]	India	2.5–5	-	1076	4	SBP: −24.27 (*p* < 0.0001) DBP: −13.28 (*p* < 0.0001) HR: −4.87 (*p* < 0.0001)	Edema: 1.77%

SESA-IVA (2007) [24]	India	2.5–5	-	30	4	2.5 mg: 150.48/92.28 to 128.57/80.86 5 mg: 165.78/95.55 to 132/82.22 *p* < 0.0001 for both	None

Bobroff et al. (2007) [57]	Ukraine	2.5–10	Amlo (5–10)	60/38	12	SBP: −30.7 versus −29.2 DBP: −13.7 versus −14.0 Target of <140/90: 83.3% versus 84.2% In both groups, significant reduction in Average daily BP Daytime BP Nighttime BP	Peripheral edema; 1.6% versus 7.8%

Basu (2007) [58]	India	5	Amlo (10)	10/10	4	SBP: 154.4 to 1304 versus 157.6 to 132.4 DBP: 99.1 to 77.5 versus 87.2 to 78.4 Equal efficacy in Daytime MAP Nighttime MAP	None

Sierkova et al.(2009) [59]	Ukraine	NA	Amlo	31/32	NA	Equal BP reduction with 2 times lower dose of S-AM	Lesser with S-AM

Koval et al. (2013) [18]	Russia	NA	Lercani	NA	NA	Similar efficacy in reducing BP in HTN with obesity	Lower with lercani

Mohanty et al. (2016) [25]	India	2.5–5	Amlo (5–10) and CLD (10–20)	60/60/60	12	NR	Incidence of Edema M: 6.7% vs 36.7% and 0.0% F: 10.0% vs 43.3% and 3.3%

AEs: adverse effects, Amlo: racemic amlodipine, BP: blood pressure, CLD: cilnidipine, DBP: diastolic BP, F: females, HR: heart rate, HTN: hypertension, Lercani: lercanidipine, M: males, MAP: mean arterial pressure, NA: not available, RRR: relative risk reduction, S-AM: S-amlodipine, SESA: safety and efficacy of S-Amlodipine, and SBP: systolic BP.

**Table 3:** Meta-analyses of S-amlodipine in hypertension.

Author (year)	Country	S-AM (mg)	Comparator (mg)	*n*	Duration (weeks)	Antihypertensive efficacy	AEs
Liu et al. (2010) [30]	China	2.5	Amlo (5)	15 trials	4–40	All trials: Similar efficacy Only high-quality trials: WMD for decrease in SBP/DBP at 4 weeks: −2.84/−1.71 8 weeks: −1.38/−1.33	Similar; RD: all trials: −0.04; High quality: −0.04

Zhao and Chen (2015) [31]	China	NA	Amlo	8 trials: 732/724	NA	Significantly better efficacy of S-AM than Amlo: OR 2.19 (*p* < 0.01)	Significantly lower rate of AEs: OR 0.51 (*p* < 0.01)

AEs: adverse effects, Amlo: racemic amlodipine, BP: blood pressure, DBP: diastolic BP, NA: not available, OR: odds ratio, RD: risk difference, S-AM: S-amlodipine, and SBP: systolic BP.

**Table 4:** Pleiotropic effects of S-amlodipine.

Author (year)	Country	S-AM (mg)	Comparator (mg)	*n*	Duration (weeks)	Antihypertensive efficacy	Pleiotropic effect
Effect on Arterial stiffness and Endothelial function							

*RCTs*							

Liangjin et al. (2013) [33]	China	2.5–5	Nifed-SR (10)	40/40	12	SBPVR (mmHg) S-AM: 14.7 ± 3.1 to 12.1 ± 2.7 (*p* < 0.05) Nifed-SR: 14.8 ± 2.9 to 13.7 ± 3.2 (*p* > 0.05) DBPVR (mmHg) S-AM: 10.2 ± 1.8 to 8.5 ± 1.9 (*p* < 0.05) Nifed-SR: 10.2 ± 1.9 to 9.8 ± 2.5 (*p* > 0.05)	CIMT (per mm) S-AM: 1.24 ± 0.41 to 1.08 ± 0.28 (*p* < 0.05) Nifed-SR: 1.23 ± 0.31 to 1.22 ± 0.33

Zhang et al. (2003) [34]	China	2.5	Amlo (5)	60	6	NA	FMD% S-AM: 4 ± 4 to 3 ± 4 (*p* = 0.01) Amlo: 6.7% to 6.8% (*p* = 0.01)

Guo et al. (2012) [35]	China	NA	Amlo	126/106	24	S-AM: 153.88/94.03 to 132.59/81.96 (*p* < 0.001 for both) Amlo: 152.21/93.3 to 133.22/82.47 (*p* < 0.001 for both)	Significant improvements in central BP components baPWV ambulatory arterial stiffness index variability of ambulatory BP (all *P* < 0.0001) CIMT: No significant changes

Si et al. (2014) [36] [crossover trial, 2-week washout]	China	2.5	Amlo (5)	24	6 × 6	SBP: 162 to 132 (Amlo) and 131 (S-AM) (*p* < 0.01 for both) DBP: 95 to 81 (Amlo) and 82 (S-AM) (*p* < 0.01 for both) HR: 76 to 72 (Amlo) and 73 (S-AM) (*p* < 0.05 for both)	FMD%: 5.7 to 8.0 (Amlo) and 7.3 (S-AM) (*p* < 0.01 for both) NMD%: 13.6 to 12.9 (Amlo) and 14.1 (S-AM) NO *μ*mol/L: 42 to 62 (Amlo) and 59 (S-AM) (*p* < 0.01 for both) eNOS *μ*/L: 20 to 26 (Amlo) and 24 (S-AM) (*p* < 0.01 for both)

*Observational study*							

Nestorovich (2013) [37] [S-AM + Enalapril versus enalapril]	Ukraine	5–10	E (10–20)	33/32	24	NR	Combination therapy had greater changes in Maximal speed (*V*max) of bloodstream in BA (i) initial (22.8 and 17.6 cm/sec) (ii) after reactive hyperaemia (41.7 and 31.6 cm/sec), Speed of retrograde wave: (i) initial (19.6 and 14.3 cm/sec) (ii) after reactive hyperaemia (25.9 and 20.2 cm/sec) (iii) post-occlusive dilatation (5.2% and 3.4%) Changes in endothelin-1 levels (i) Combination: 3.86 ± 0.24 to 1.95 ± 0.19 pg/mL, *p* < 0.05 (ii) Enalapril alone: 3.32 ± 0.27 to 1.83 ± 0.21 pg/mL, *p* < 0.05

*Effect on LV and BA function *							

Iskenderov and Saushkina (2013) [38]	Russia	-	Amlo	61/66	24	Comparable BP reduction at lower dose of S-AM	S-AM was associated with - Complete regression of LVH: 51% cases Normalization of LV diastolic function: 62.4% cases Significant improvement in BA vasomotor function Significant reduction in atherogenic lipoproteins and TC

*Efficacy in renal transplant cases*							

Tang et al. (2003) [39]	China	2.5–5	Amlo	20	8	Significant reduction in SBP (*p* < 0.01) DBP (*p* < 0.01) BUN (*p* < 0.05)	Normalization of BP in 85% cases Improved renal function

*Effect on insulin resistance [RCT]*							

Xiao et al. (2016) [40]	China	2.5–5	Losartan (50–100)	112/115	156	BP reduction was significant and similar in both groups	In both groups, significant reduction in fasting insulin Increase in insulin sensitivity index

*Effect on platelet aggregation and expression of MMP 2 and MMP 9*							

Li et al. (2013) [41]	China	NA	-	32	NA	NA	Reduced platelet aggregation maximal (%): 47.77 ± 11.92 to 40.78 ± 13.97 (*p* < 0.05) Platelet inhibition rate (%): 13.5 ± 25.23 No effect on MMP levels

AEs: adverse effects, Amlo: racemic amlodipine, BA: brachial artery, baPWV: brachial artery pressure wave velocity, BP: blood pressure, BUN: blood urea nitrogen, CIMT: carotid intima media thickness, DBP: Diastolic BP, DBPVR: DBP variety ratio, eNOS: endothelial nitric oxide synthase, FMD: flow-mediated dilation, HR: heart rate, LV: left ventricle, LVH: left ventricular hypertrophy, MMP: matrix metalloproteinase, NA: not available, Nifed-SR: nifedipine sustained release, NMD: nitroglycerine-mediated dilatation, NO: nitric oxide, NR: not reported, OR: odds ratio, RD: risk difference, S-AM: S-amlodipine, SBP: systolic BP, SBPVR: SBP variety ratio, and TC: total cholesterol.
